# Integrative Principal Component–QTL Mapping Identifies Genetic Modifiers of Tumor and Metabolic Traits in *Smad4*-Deficient Collaborative Cross Mice

**DOI:** 10.3390/ijms27167254

**Published:** 2026-08-14

**Authors:** Osayd Zohud, Kreem Midlej, Fuad A. Iraqi

**Affiliations:** Department of Clinical Microbiology and Immunology, Gray Faculty of Medicine and Health Sciences, Tel-Aviv University, Tel Aviv-Yafo 6997801, Israel; osaydzohud@mail.tau.ac.il (O.Z.); kareemmidlej@mail.tau.ac.il (K.M.)

**Keywords:** Collaborative Cross mice, *Smad*4, QTL mapping, principal component analysis, intestinal tumorigenesis, systems genetics, genetic modifiers, sex-specific effects

## Abstract

Genetic background strongly influences the penetrance and phenotypic expression of SMAD4-associated intestinal tumorigenesis, yet the underlying modifier loci remain poorly defined. To investigate the genetic architecture of tumor susceptibility and systemic physiology, we analyzed 260 *Smad4*^+^/^−^ × Collaborative Cross (CC)-F1 mice derived from 14 CC lines using 11 quantitative traits, including longitudinal body weight, adjusted organ weights, and intestinal polyp counts across anatomical regions. Principal component analysis reduced these traits to seven components explaining more than 85% of total phenotypic variance. PC1 represented a tumor burden–metabolic axis, whereas PC2 captured systemic organ-physiology variation. Genome-wide QTL mapping of principal component scores identified significant loci for PC1 on chromosomes 1 and 4 and a female-specific locus for PC5 on chromosome 10, with additional suggestive loci supporting a polygenic architecture. Founder-effect analysis revealed strong contributions from CAST/EiJ, 129S1/SvImJ, and WSB/EiJ haplotypes. Candidate gene annotation identified biologically relevant coding and noncoding loci, including *Galnt7* and *Galntl6*, as well as regulatory regions with potential enhancer activity. Together, these findings indicate that intestinal tumor susceptibility in *Smad4*^+^/^−^ × CC-F1 mice is influenced by multiple coding and regulatory genetic modifiers with sex-dependent effects. This study demonstrates that integrating multivariate phenotyping with systems genetics analyses provides an effective framework for identifying the complex genetic networks underlying intestinal tumorigenesis and associated systemic physiological variation in genetically diverse mouse populations.

## 1. Introduction

Grasping how complex traits—especially those linked to cancer risk and overall body function—are shaped by genetics remains a major challenge in mammalian biology. These traits usually involve multiple genes and interact through connected biological systems, meaning that changes in one area, such as metabolism, can impact others, such as tumor growth, due to shared genetic pathways [1]. To make sense of this complexity, scientists need both genetically varied populations and data analysis tools that can simplify large sets of biological data into meaningful patterns [2].

One powerful resource for investigating complex genetic traits is the Collaborative Cross (CC) mouse panel, a genetically diverse reference population of recombinant inbred mouse strains derived from eight genetically distinct founder strains, including five classical laboratory strains (A/J, C57BL/6J, 129S1/SvImJ, NOD/ShiLtJ, and NZO/HlLtJ) and three wild-derived strains (CAST/EiJ, PWK/PhJ, and WSB/EiJ) [3]. The CC was specifically developed to model the extensive genetic diversity observed in natural populations while maintaining the reproducibility afforded by inbred lines. This design captures about 90% of the genetic diversity found in the species *Mus musculus*, thereby generating substantial variation in disease susceptibility, immune function, metabolic phenotypes, and other complex traits that more closely resemble the genetic heterogeneity observed in humans. Unlike traditional two-parent crosses, CC mice provide greater genetic resolution, balanced representation of founder alleles, and enhanced statistical power to detect quantitative trait loci (QTLs), epistatic interactions, and genetic modifiers underlying complex diseases [4]. These characteristics make the CC an ideal platform for investigating how host genetic background influences the penetrance and phenotypic variability of Smad4-associated intestinal tumorigenesis.

Juvenile Polyposis Syndrome (JPS) is a genetic disorder usually caused by mutations in the *Smad4* gene, which plays a key role in the TGF-β signaling pathway. The marked phenotypic variability observed among individuals carrying the same *Smad4* mutation makes JPS a valuable model for investigating how genetic background influences disease susceptibility and progression. People with *Smad4* mutations can experience a wide range of symptoms, including differences in the number and location of polyps and the risk of developing cancer, even among individuals with the same mutation [5,6]. This suggests that other genes in a person’s background may influence how the disease appears. This phenomenon is consistent with the action of genetic modifier genes, which are secondary loci that alter the penetrance, severity, or clinical presentation of a primary disease-causing mutation. Modifier effects have been recognized in numerous Mendelian disorders and are thought to contribute to the marked phenotypic heterogeneity observed among individuals with JPS carrying the same *Smad4* mutation. Identifying these modifiers is therefore essential for understanding the biological mechanisms underlying disease variability and colorectal cancer susceptibility [7,8]. To study this, we have bred *Smad4* mutations into CC mice and found that different genetic backgrounds lead to varying tumor patterns, sizes, and related immune and metabolic traits [9]. Our previous work has shown that different genetic backgrounds lead to varying tumor patterns, sizes, and related immune and metabolic traits [9,10]. These observations support the hypothesis that multiple genetic modifiers contribute to cancer susceptibility and that the host genetic background plays a critical role in determining disease severity.

Looking beyond just polyp counts, other research shows that cancer development interacts with whole-body traits like body weight, organ size, and metabolism [11]. This coordinated influence on multiple phenotypic traits is consistent with the concept of pleiotropy, whereby a single gene or genetic locus affects two or more seemingly distinct traits. Pleiotropy is a fundamental principle in quantitative genetics and is particularly relevant to complex diseases such as cancer, where shared genetic pathways can simultaneously influence tumor development, metabolism, immune responses, and other physiological processes. Consequently, evaluating these traits jointly may provide a more comprehensive understanding of the genetic architecture underlying disease susceptibility [12,13]. In fact, CC and related mouse populations have helped map genes involved in glucose regulation, body composition, and energy balance [14]. These studies reveal new genes and confirm previously identified ones, showing that genetics influences both metabolism and disease risk. However, few studies have fully explored how these systems interact with tumor development in a single, integrated analysis [15,16,17].

Our study builds on previous work by using a combined, multivariate method to explore how genetics affects both intestinal tumors and broader physiological traits in *Smad4*-deficient CC mice [10]. We collected data on 11 quantitative phenotypic measurements, including body weight over time (up to 80 weeks), organ weights (like liver, spleen, kidneys, heart, and brain), and polyp counts in different regions of the intestine (duodenum, jejunum, ileum, and colon). These traits span metabolic, brain-related, immune, and cancer-related systems.

To manage the complexity of this data, we used Principal Component Analysis (PCA), a statistical method that simplifies high-dimensional data by identifying key patterns, called principal components [18]. These components summarize variation across multiple correlated phenotypic measurements, enabling the identification of genetic regions (QTLs) with potential pleiotropic effects that might be overlooked when each phenotype is analyzed individually. This approach has worked well in other genetics studies, where it helps uncover networks of traits that are controlled together [19].

In this study, we used PCA to integrate tumour-related and systemic physiological measurements in *Smad4*-mutant CC mice. This approach identified seven principal components that captured most of the observed phenotypic variation and enabled the mapping of associated QTLs. We identified sex-specific genetic loci and biologically plausible candidate genes, including *Galnt7*, providing new insights into the complex genetic architecture underlying intestinal tumorigenesis and systemic physiological variation.

By applying this multivariate method, our work offers a new way to explore how genetic diversity shapes both disease and overall biology, helping to reveal the complex genetic networks behind cancer and other interconnected traits.

## 2. Results

### 2.1. Principal Component Analysis Reveals Latent Structure in Tumor and Organ Phenotypes

To better understand how the different phenotypes relate to one another, we applied principal component analysis (PCA) to summarize variation across all the measured traits, including body weight changes, organ weights, and intestinal polyp counts. Together, the first seven principal components explained approximately 85% of the total phenotypic variation among *Smad*4^+^/^−^ × CC-F1 mice. The first principal component (PC1), accounting for 31.2% of the variance, described a clear relationship between overall body weight trajectory and polyp burden across the intestinal tract. Mice with higher polyp counts tended to show distinctive growth patterns, suggesting that PC1 represents a combined tumor load–metabolic axis. The second component (PC2), explaining 26.1% of the variance, was largely shaped by variation in organ weights—particularly those of the liver, kidney, spleen, and brain—indicating a systemic physiological gradient among the lines. The remaining components (PC3–PC7) accounted for more specific differences, such as the distribution of polyps along different intestinal regions or the scaling of individual organs with overall body mass (Supplementary Figure S1).

When the analysis was performed separately for males and females, broadly similar component structures were observed, although the relative contributions of individual traits differed between the sexes. In males, the first principal component (Supplementary Figure S2) was primarily driven by Δ body weight % and intestinal polyp counts, defining a strong tumor load–metabolic axis. In contrast, in females (Supplementary Figure S3), the first axis was also dominated by intestinal traits, but the second component captured more variance from organ weights, particularly those of the liver and kidneys. These patterns suggest that, although both sexes share a common relationship between body weight and tumor burden, females exhibit additional variation in organ physiology, reflecting sex-dependent differences in phenotypic integration.

### 2.2. QTL Mapping Using Principal Component Scores Identifies Multiple Significant Loci

Principal component analysis (PCA) was used to reduce multiple correlated phenotypic traits—body weight profiles, adjusted organ weights, and polyp counts—into composite variables representing major axes of biological variation. Seven principal components (PCs) were retained, together explaining over 85% of the total variance.

Genome-wide QTL mapping was then performed using each PC score as a quantitative phenotype to identify genomic loci contributing to systemic and intestinal variation.

#### 2.2.1. Significant QTL at the Genome-Wide *p* < 0.05 Level

**Figure 1:**
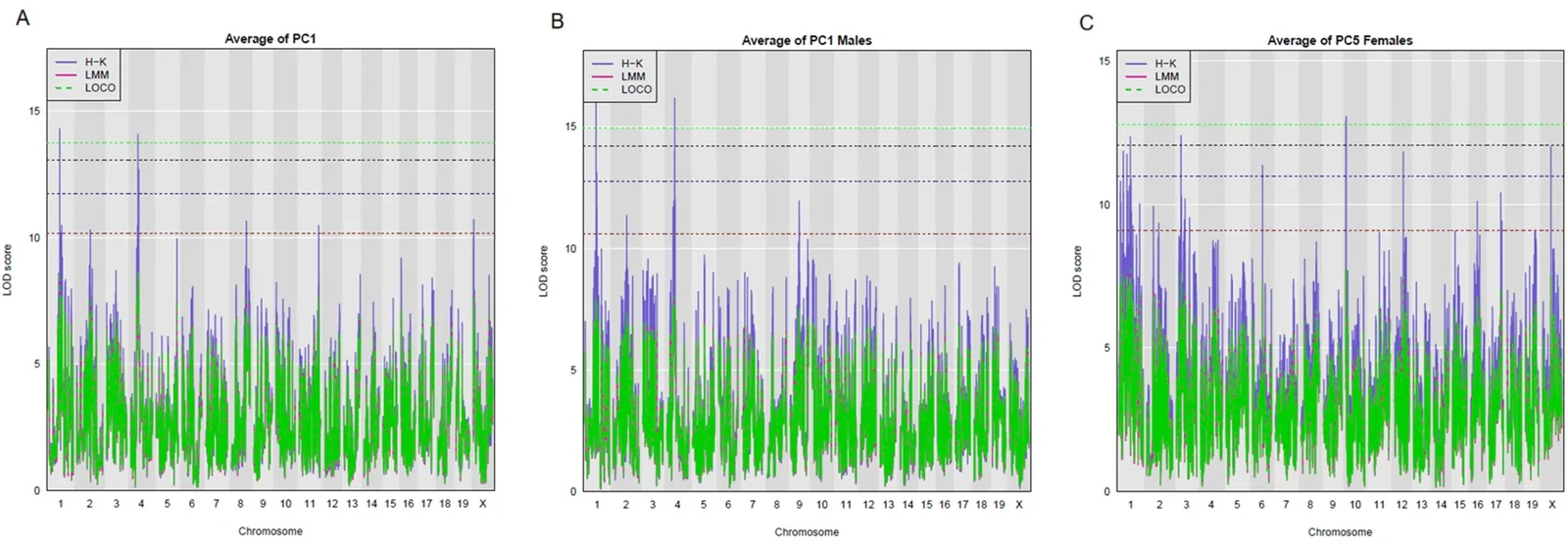
Genome-wide QTL mapping for principal components derived from phenotypic traits in *Smad*4^+^/^−^ × CC-F1 mice: (**A**) QTL scan for PC1 in the combined population showing major peaks on Chromosomes 1 and 4. (**B**) Male-specific QTL scan for PC1 revealing similar loci on Chromosomes 1 and 4. (**C**) Female-specific QTL scan for PC5 identifying a significant QTL on Chromosome 10. Horizontal dashed lines represent the genome-wide significance thresholds at *p* < 0.05 (solid/top line) and *p* < 0.1 (dashed/second line), determined by 10,000 permutations. Chromosomal positions are shown along the x-axis, and LOD scores on the y-axis.

Using a permutation-derived significance threshold (genome-wide alpha = 0.05), we identified QTLs for multiple principal components (PCs) in both the combined population and sex-specific analyses (Table 1). These genome-wide significant loci were designated as intestinal polyposis susceptibility loci (*Ips*l), hereafter referred to as *Ips*l, reflecting their role in modulating variation in tumor burden and associated physiological traits across the Collaborative Cross population. In the combined dataset, the strongest signals were found for PC1, corresponding to the tumor burden–body weight axis, with major loci detected on Chromosomes 1 and 4 (Figure 1A). In male mice, significant QTLs for PC1 were localized on Chromosomes 1 and 4 (Figure 1B), mirroring the associations identified in the combined cohort and reinforcing the genetic contribution to tumor–metabolic coupling. In females, a distinct QTL for PC5 was identified on Chromosome 10 (Figure 1C), suggesting sex-dependent genetic modulation of traits related to organ scaling and regional polyp distribution. Collectively, these loci indicate that multiple, partially overlapping genetic factors underlie systemic physiology and intestinal tumor susceptibility across both sexes.

#### 2.2.2. Suggestive QTL at the Genome-Wide *p* < 0.1 Level

**Figure 2:**
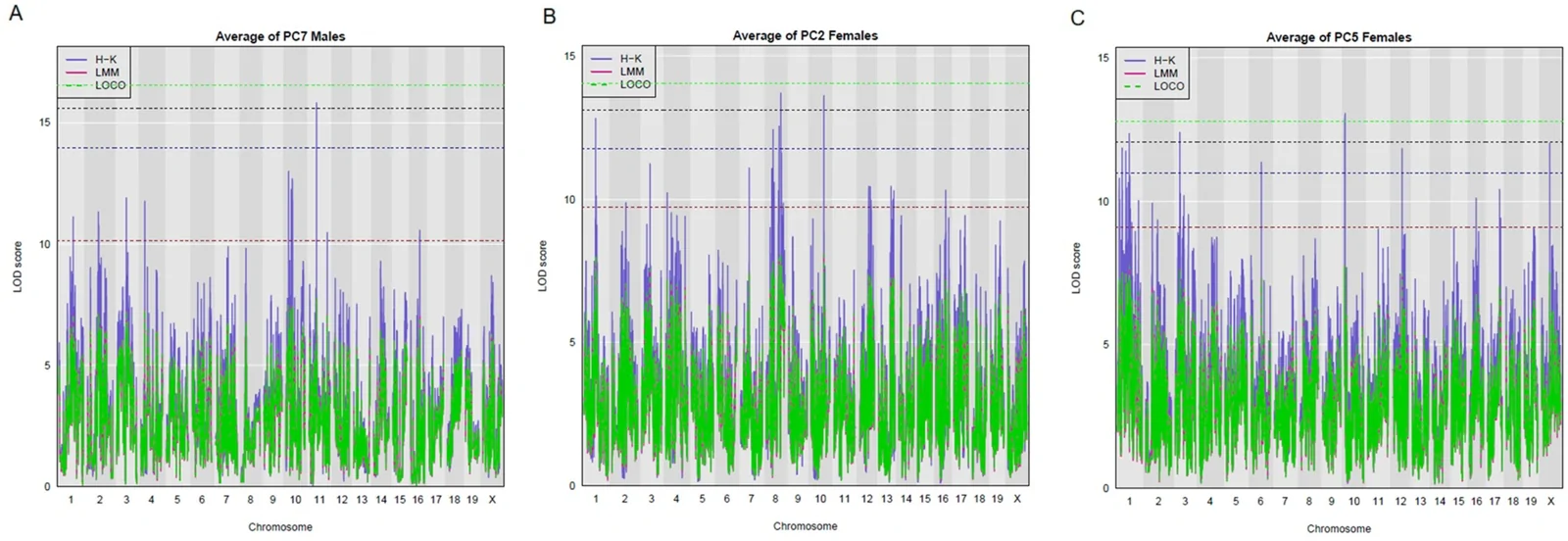
Suggestive QTLs (*p* < 0.1) identified for principal components in *Smad*4^+^/^−^ × CC-F1 mice. Genome-wide QTL scans illustrating loci exceeding the 0.1 suggestive threshold but below the 0.05 genome-wide significance cutoff. Horizontal dashed lines represent the genome-wide significance thresholds at *p* < 0.05 (solid/top line) and *p* < 0.1 (dashed/second line), determined by 10,000 permutations. These loci indicate additional genomic regions that may be involved in the secondary modulation of body weight, organ scaling, and intestinal tumor burden: (**A**) PC7 in males, reflecting minor QTL signals associated with coordinated variation in visceral organ weights. (**B**) PC2 in females, showing suggestive loci associated with variation in organ-weight traits. (**C**) PC5 in females, revealing loci linked to regional polyp distribution and organ-scaling effects. Chromosomal position (Mb) and LOD score are plotted on the x- and y-axes, respectively.

Applying a more relaxed suggestive threshold (genome-wide alpha = 0.1), as detailed in Table 2, we identified additional loci across PCs 1–6 (Figure 2), consistent with polygenic regulation of complex traits.

Although these signals explained smaller proportions of variance than the genome-wide significant QTLs, they highlight genomic regions potentially involved in the secondary modulation of body weight, organ size, and polyp number.

These loci provide promising targets for further investigation and may represent modifier regions that fine-tune the major tumor–metabolic effects identified at the 0.05 threshold.

#### 2.2.3. Potential Loci at the Genome-Wide *p* < 0.5 Level

To capture the broader landscape of genetic variation, loci detected at a permutation-based genome-wide threshold of *p* < 0.5 are reported in Supplementary Table S1. While these loci do not meet the stringent criteria for genome-wide significance or suggestiveness, they provide supporting evidence for minor-effect regions and potential additive or epistatic interactions contributing to the observed phenotypic diversity.

### 2.3. Founder Effects Across Significant and Suggestive QTLs

**Figure 3:**
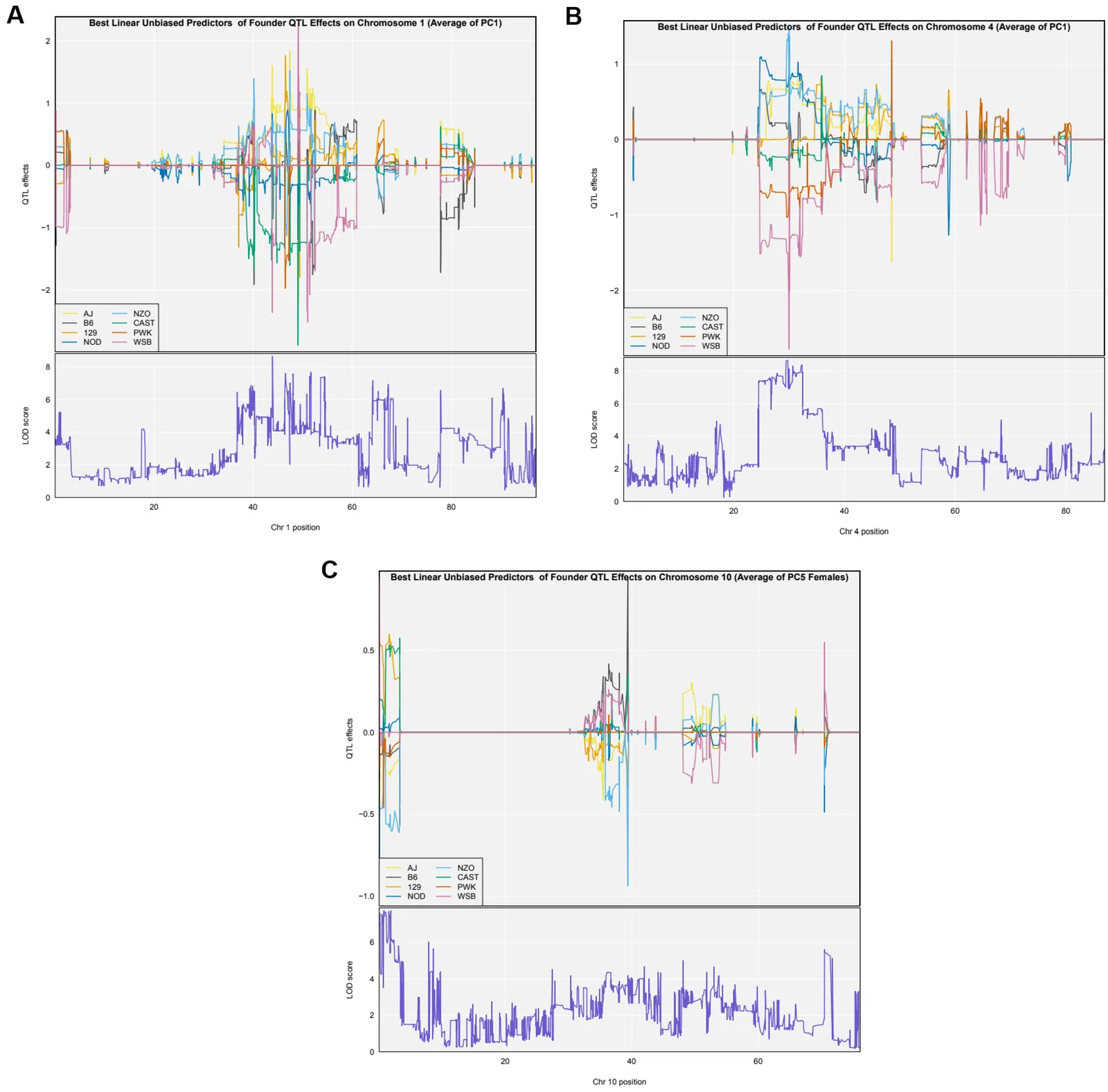
Founder-effect estimates for significant QTLs detected at the 0.05 significance level: (**A**) Founder allelic effects at the Chr1 QTL peak for PC1 (combined population + males). (**B**) Founder effects at the Chr4 QTL peak for PC1 (combined population + males). (**C**) Founder effects at the Chr10 QTL peak for PC5 (females). Each colored line represents one of the eight CC founder strains (A/J, C57BL/6J, 129S1/SvImJ, NOD/ShiLtJ, NZO/HlLtJ, CAST/EiJ, PWK/PhJ, WSB/EiJ). The y-axis indicates the estimated additive effect on the respective PC value, with positive values denoting alleles associated with increased trait expression. The lower figures with the blue line in (**A**,**B**) show the location of the QTL peaks.

Founder-effect analysis of genome-wide significant QTLs (Figure 3A–C) revealed distinct allelic contributions among Collaborative Cross founder strains, highlighting diverse genetic influences on the integrated phenotypic axes represented by the principal components. For PC1, which captured the tumor burden–body weight relationship, two major loci were identified on Chromosomes 1 and 4. The Chromosome 1 locus (*IPSL1*; 43.91 Mb) was primarily driven by a strong positive allelic effect from CAST/EiJ, suggesting that CAST alleles enhance traits associated with increased intestinal tumor load and altered metabolic status. In contrast, the Chromosome 4 locus (*IPSL14*; 29.77 Mb) was dominated by a positive effect from 129S1/SvImJ, indicating a distinct genetic background influencing the same phenotypic axis. These findings imply that at least two founder-derived haplotypes contribute independently to systemic metabolic–tumor interactions in the *Smad4*^+/−^ × CC-F1 population.

A female-specific QTL for PC5 on Chromosome 10 (*IPSL54*; 1.93 Mb) revealed further allelic diversity, with a positive contribution from WSB/EiJ and a negative effect from CAST/EiJ. This contrast suggests that WSB alleles promote increased organ-weight scaling and intestinal morphological variation in females, while CAST alleles exert a suppressive effect on these traits. The absence of notable male-specific founder deviations at other loci reinforces the observation that sex-dependent genetic modulation is more pronounced for organ and intestinal traits in females.

**Figure 4:**
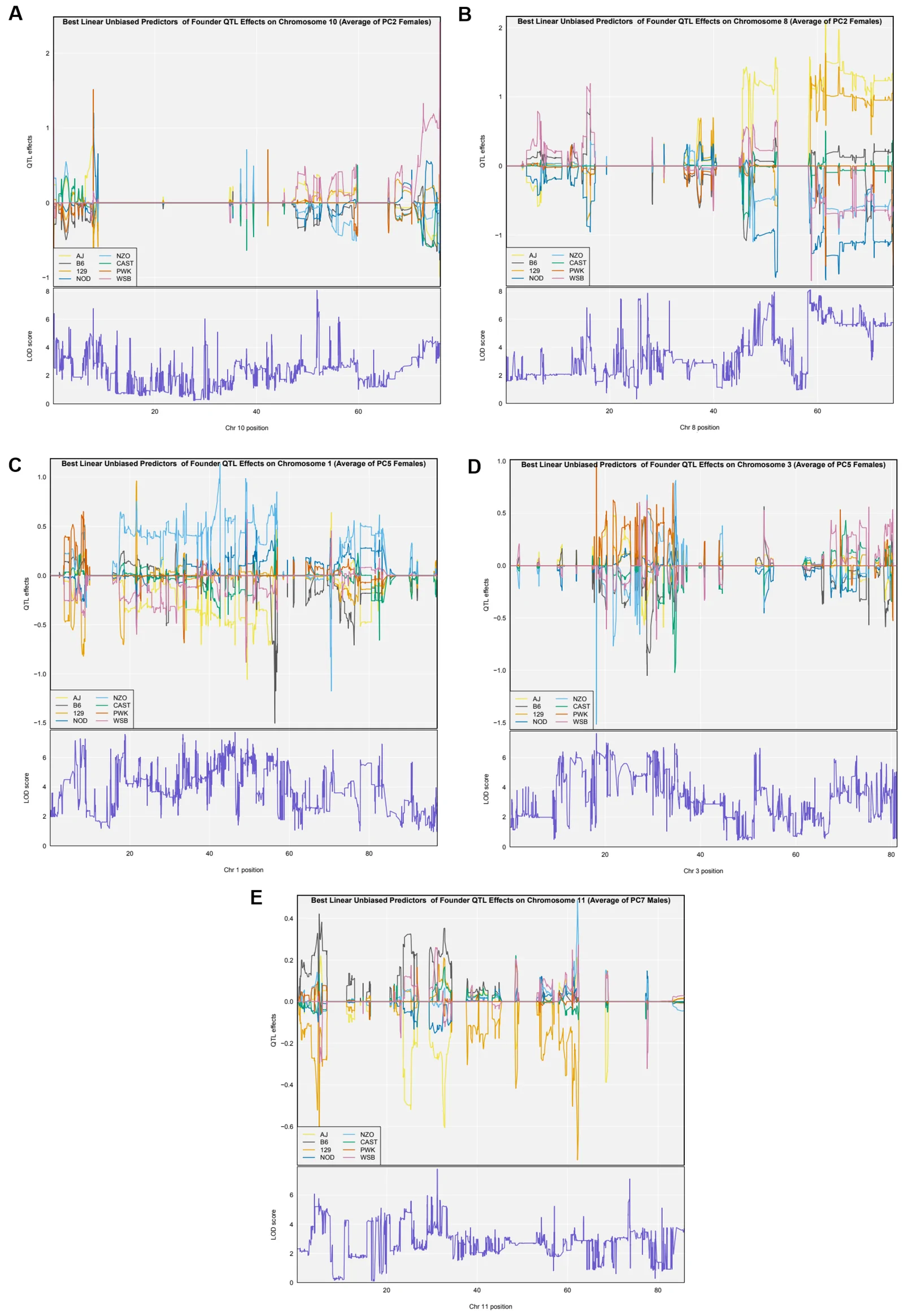
Founder-effect profiles for suggestive QTLs (*p* < 0.1). Founder-effect plots illustrating the allelic contributions of the eight Collaborative Cross founder strains (A/J, C57BL/6J, 129S1/SvImJ, NOD/ShiLtJ, NZO/HlLtJ, CAST/EiJ, PWK/PhJ, and WSB/EiJ) for loci detected at the 0.1 significance threshold: (**A**) Female PC2 locus on Chromosome 10 showing positive effects from C57BL/6J and NZO/HlLtJ and negative effects from CAST/EiJ and PWK/PhJ. (**B**) Female PC2 locus on Chromosome 8 exhibiting a similar founder pattern with moderate allelic variation. (**C**) Female PC5 locus on Chromosome 1 with positive effects from 129S1/SvImJ and PWK/PhJ founders and negative effects from C57BL/6J and CAST/EiJ. (**D**) Female PC5 locus on Chromosome 3 showing comparable directional effects among founder strains. (**E**) Male PC7 locus on Chromosome 11 displaying positive contributions from WSB/EiJ and NZO/HlLtJ and negative effects from CAST/EiJ and PWK/PhJ. Positive values correspond to alleles that increase the respective principal-component score, indicating founder backgrounds that contribute to secondary phenotypic variation related to organ scaling, systemic physiology, and tumor-load modulation. The lower figures with the blue line in (**A**–**E**) show the location of the QTL peaks.

Founder-effect analyses of the suggestive QTLs identified at the 0.1 LOD threshold revealed distinct yet modest allelic contributions across multiple chromosomes, consistent with polygenic regulation of complex physiological traits (Figure 4). In female mice, loci on Chromosomes 1 (*IPSL53*; 46.35 Mb) and 3 (*IPSL65*; 18.2 Mb) associated with PC5 displayed positive founder effects from 129S1/SvImJ and PWK/PhJ, with corresponding negative effects from CAST/EiJ and C57BL/6J, suggesting that these alleles may influence organ-weight scaling and regional intestinal features in a sex-dependent manner. A second female-specific QTL on Chromosome 8 (*IPSL33*; 58.6 Mb), linked to PC2, showed moderate positive contributions from CAST/EiJ and negative effects from PWK/PhJ, indicating the involvement of distinct founder haplotypes in modulating systemic physiological variation. Similarly, a locus on Chromosome 10 (IPSL17; 51.83 Mb) exhibited a primarily positive deviation for WSB/EiJ, suggesting a limited but detectable founder influence on traits captured by PC2 in females.

In males, a QTL on Chromosome 11 (*IPSL82*; 31.2 Mb) associated with PC7 showed subtle founder differentiation, with CAST/EiJ contributing weakly positive effects and PWK/PhJ displaying minor negative deviations, while the remaining founders clustered near zero. This pattern, together with the low overall amplitude of allelic effects, suggests that these loci represent modest, polygenic influences on male-specific physiological variance. Collectively, the founder-effect profiles across these suggestive loci reinforce the interpretation that secondary QTLs reflect distributed allelic effects rather than single, dominant haplotypes—an observation consistent with previous findings in Collaborative Cross studies of multigenic traits.

### 2.4. Candidate Genes Within Significant QTL Intervals

Annotation of the significant QTL intervals revealed both protein-coding and noncoding loci, reflecting a combination of structural and regulatory contributions to the phenotypic variation observed across Collaborative Cross backgrounds. The integration of these genetic and functional data supports a multifactorial model of phenotypic regulation involving both enzymatic activity and transcriptional control.

The most functionally rich locus was identified on Chromosome 8 (*IPSL33*; 58.10–58.64 Mb), corresponding to PC2 in female mice. This interval harbors two well-characterized genes, *Galnt7* and *Galntl6*, both belonging to the polypeptide N-acetylgalactosaminyltransferase (GALNT) family. These enzymes catalyze the first step in mucin-type O-glycosylation—a post-translational modification essential for epithelial barrier formation and mucosal homeostasis [20]. Experimental evidence links *GALNT7* dysregulation to epithelial proliferation, immune modulation, and colorectal cancer progression [21,22]. The presence of these genes within a sex-specific QTL associated with systemic physiological traits suggests that variation in glycosylation pathways may underlie differential tumor susceptibility and organ physiology in females. Several nearby predicted transcripts (*Gm45644*, *Gm45359*, *Gm45633*) lack experimental characterization but may represent local regulatory elements.

In contrast, the Chromosome 10 QTL (*IPSL17*; 51.83 Mb) contains a single predicted noncoding transcript, *Gm40646*, annotated in Ensembl but without known function. FANTOM5 and Ensembl expression data indicate low-level transcriptional activity in hepatic and intestinal tissues. Based on its genomic context and lack of protein-coding potential, *Gm40646* is most likely a long noncoding RNA (lncRNA) with potential cis-regulatory effects on nearby genes, consistent with the organ-weight and metabolic traits loading on PC5 [23].

Several other QTLs correspond to noncoding or enhancer-rich regions, particularly those on Chromosome 4. The loci at 8.17–8.24 Mb and 29.37–30.67 Mb include *Gm11923* and *4930556G01Rik*, both annotated as noncoding transcripts supported by RNA-seq data. According to the FANTOM5 enhancer atlas [24], this region overlaps transcriptionally active enhancer domains marked by H3K27ac and H3K4me1 modifications. These features suggest that cis-regulatory variation may influence local or distal gene expression, thereby modulating systemic and intestinal traits captured by PC1.

The Chromosome 1 QTL (*IPSL1*; 43.86–43.95 Mb) lies in an intergenic region devoid of annotated genes but rich in open-chromatin and enhancer-associated histone marks. This pattern is consistent with a noncoding regulatory locus, similar to enhancer-linked QTLs described in other Collaborative Cross studies [17,25]. Such loci may regulate distant genes involved in metabolism or tumor suppression, potentially through long-range chromatin interactions.

Overall, these findings reveal that phenotypic axes such as tumor burden, metabolic rate, and organ physiology are influenced by both protein-coding genes (e.g., *Galnt7*, *Galntl6*) and noncoding regulatory elements (*Gm11923*, *Gm40646*, *4930556G01Rik*). This highlights the central role of noncoding genetic variation in shaping complex phenotypes in genetically diverse populations.

## 3. Discussion

This study introduces a comprehensive multivariate approach to investigate the genetic basis of intestinal tumor susceptibility and systemic physiological traits in Smad4^+^/^−^ × Collaborative Cross (CC) F1 mice. By integrating Principal Component Analysis (PCA) with genome-wide QTL mapping, we identified both coding and noncoding genomic regions that contribute to coordinated phenotypic patterns involving tumor load, metabolism, and organ structure. These results build upon prior research on Smad4-driven intestinal disease, demonstrating that the variability in polyposis severity is shaped by a distributed network of genetic modifiers, rather than any single locus.

### 3.1. Multivariate Trait Integration and Genetic Architecture

PCA enabled us to reduce complex and interrelated traits into biologically meaningful composite variables. The first principal component (PC1) captured a strong correlation between polyp burden and body weight progression, defining a “tumor load–metabolic axis,” while PC2 reflected variability in organ weights (particularly the liver, kidney, and spleen). These patterns align with recent systems genetics studies emphasizing the value of multivariate analysis in uncovering pleiotropic effects in complex traits [26,27]. Rather than mapping each trait in isolation, analyzing PCs revealed loci likely to regulate interconnected physiological processes like energy homeostasis, growth, and gut maintenance [1].

### 3.2. Polygenic Modulation of Tumor and Systemic Traits

Genome-wide QTL mapping revealed significant loci at the 0.05 threshold, including key signals on Chromosomes 1 and 4 (PC1), as well as a female-specific QTL on Chromosome 10 (PC5). These findings support a polygenic model of tumor–metabolic coupling in *Smad4*^+^/^−^ × CC-F1 mice, with contributions from multiple founder alleles. In particular, alleles from CAST/EiJ and 129S1/SvImJ strains were most strongly associated with tumor-related traits, consistent with prior studies linking these strains to epithelial proliferation and inflammatory responses [28]. The female-specific QTL on Chromosome 10 highlights growing recognition of sex-specific genetic influences on cancer susceptibility and metabolic regulation [29]. This sex-based variation likely reflects differences in hormonal and immune signaling that interact with *Smad*4 and epithelial maintenance [30].

Additional loci identified at the 0.1 threshold, while individually weaker, still provide meaningful insight into the polygenic nature of these traits. The dispersed effects across multiple founder alleles suggest a model of genetic fine-tuning rather than singular major-effect loci—echoing results from other CC-based trait mapping efforts [17,25].

### 3.3. Functional and Regulatory Implications of Candidate Loci

Our candidate gene analysis uncovered both structural and regulatory contributors. On Chromosome 8 (IPSL33), we identified Galnt7 and Galntl6, genes encoding O-glycosyltransferases involved in mucin production and epithelial barrier function [20]. Aberrant *Galnt7* expression has been implicated in colorectal tumor progression and immune dysregulation [21,22], suggesting that natural variation in glycosylation pathways may influence epithelial integrity and polyp development in a genotype-dependent manner.

In contrast, QTLs on Chromosomes 1 and 4 were located in gene-sparse or noncoding regions enriched with enhancer-associated histone modifications (H3K4me1, H3K27ac), supporting a regulatory rather than structural function [31,32]. Notably, these regions contained long noncoding RNA (lncRNA) candidates such as Gm11923 and 4930556G01Rik. This is consistent with growing evidence that noncoding elements play essential roles in regulating tissue-specific gene expression networks involved in metabolism and immunity [23]. Together, these findings emphasize the importance of regulatory variation—not just protein-coding changes—in shaping phenotype.

### 3.4. Sex-Specific and Systemic Regulation

The detection of female-specific QTLs reinforces the need to treat sex as a fundamental biological variable in genetic studies. Distinct founder effects in females, especially for PC5 and PC2, suggest that hormonal or epigenetic regulation may interact with genetic variation to modulate systemic physiology. Previous CC studies have observed similar sex-specific effects in metabolic and inflammatory phenotypes [16,33]. The alignment of tumor, metabolic, and organ weight traits along shared PCA dimensions supports a model in which common genetic regulators control systemic balance and epithelial maintenance—possibly through pathways involving TGF-β/Smad4 signaling [34,35].

### 3.5. Strengths, Limitations, and Future Directions

A key strength of this study is the integration of multivariate phenotype analysis with systems-genetic mapping in a genetically diverse population, enabling discovery of pleiotropic and sex-specific QTLs that may be overlooked in univariate scans. However, the F1 cross design restricts the resolution of causal variant mapping and may limit the direct generalizability of the identified QTLs to genetically heterogeneous outbred mouse populations, and PCA-derived components may obscure individual trait effects. Furthermore, although genome-wide significance thresholds were determined empirically using permutation testing and QTL mapping was performed using an additive founder-haplotype model with LOCO correction, non-additive genetic effects, including dominance and epistasis, were not evaluated. Consequently, some genetic effects may not have been detected, whereas some identified QTLs may represent false-positive associations that require independent validation. These methodological limitations should therefore be considered when interpreting the identified QTLs and their associated candidate genes.

In addition, sex-stratified QTL analyses reduced the sample size within individual CC line-by-sex groups, which may have limited the power to detect QTLs with modest effect sizes. Consequently, larger populations will be important for validating the identified loci and further refining their genomic intervals. Moreover, candidate gene prioritization was based on positional mapping and biological plausibility rather than direct functional evidence. Therefore, the proposed candidate genes, including Galnt7, Galntl6, and the identified noncoding RNAs, should be considered putative genetic modifiers until experimentally validated. Future work should incorporate transcriptomics and chromatin accessibility assays (e.g., ATAC-seq) to confirm the functional impact of candidates like Galnt7 and Gm40646. Allele-specific expression analyses across founder lines could also clarify whether these QTLs act in cis or trans. Moreover, applying this approach to additional Smad4-related disease models (e.g., inflammation-induced CRC) may uncover conserved regulatory pathways linking epithelial homeostasis, metabolism, and cancer susceptibility.

From a translational perspective, the modifier loci identified in this study provide a valuable framework for future precision medicine research. Although therapeutic validation was beyond the scope of the present work, these QTLs represent promising targets for functional characterization using congenic mouse lines, CRISPR/Cas9-mediated genome editing, and integrative multi-omics approaches to identify the underlying causal genes and molecular mechanisms. Once validated, these genetic modifiers could be evaluated as biomarkers for disease susceptibility, tumor progression, and variability in therapeutic response. Furthermore, the identification of sex-specific QTLs suggests that host genetic background and biological sex may jointly influence treatment efficacy, supporting future investigations aimed at genetically informed and sex-aware therapeutic stratification for colorectal cancer. Collectively, these findings establish a foundation for translating genetic modifier discovery into personalized prevention and therapeutic strategies.

## 4. Materials and Methods

### 4.1. Ethics and Animal Welfare Considerations

All the animal procedures conformed to the ethical standards for the care and use of laboratory animals and were approved by the Institutional Animal Care and Use Committee (IACUC) of Tel Aviv University under authorization number 01-19-044. Mice were monitored daily for clinical signs of distress. Humane endpoints, including weight loss exceeding 20% or signs of suffering, were determined in consultation with veterinary staff.

### 4.2. Mouse Lines and Breeding Strategy

The genetically diverse Collaborative Cross (CC) mouse lines used in this study have been described previously [9,36,37]. A total of 260 F1 offspring were generated from 14 CC lines by crossing male CC mice with female C57BL/6J-*Smad4*^tm1Mak/+^ heterozygous knockout mice (Jackson Laboratory, Bar Harbor, ME, USA). The parental mice were weaned at 3–4 weeks of age and first mated at 8 weeks of age. Approximately two breeding cages were established for each CC line. The breeding scheme consisted of one CC male and two to three C57BL/6J-*Smad4*^tm1Mak/+^ females per breeding cage, as previously described [38]. The C57BL/6J-*Smad4*^tm1Mak/+^ mice carry a heterozygous targeted disruption of the *Smad4* gene, resulting in Smad4 haploinsufficiency and providing a genetically sensitized background for investigating the influence of host genetic modifiers on disease phenotypes associated with germline *Smad4* deficiency [39]. The identities of the 14 CC strains used in this study are provided in Supplementary Table S2. Detailed information on the founder haplotype composition, genetic background, and breeding history of each strain is available through the Collaborative Cross resource [9,10,40].

The resulting F1 mice were maintained under specific pathogen-free (SPF) conditions at the Small Animal Facility of the Sackler Faculty of Medicine, Tel Aviv University. Animals were housed in open-top cages with hardwood chip bedding under a 12:12 h light–dark cycle at 21–23 °C and provided ad libitum access to water and a standard chow diet (TD.2018SC; Harlan Teklad Global, Madison, WI, USA), containing 18% fat, 24% protein, and 58% carbohydrates.

### 4.3. Genotyping of F1 Mice

For genomic DNA extraction, the NaOH method was employed, as referenced in [41]. Tail samples measuring 3–4 mm were collected into Eppendorf tubes, to which a mixture of 75 µL of 25 mM NaOH and 0.2 mM EDTA was added. These samples were then heated at 98 °C for 1 h in a thermocycler, cooled to 15 °C, and neutralized with 75 µL of 40 mM Tris HCl (pH 5.5) post-heating. Centrifugation at 4000 rpm for 3 min helped clarify the samples, which were then ready for PCR genotyping.

Specific three-set primer pairs were used for PCR-based genotyping:

- Primer 30403 (5′-TGT AGT TCT GTC TTT CCT TCC TG-3′).
- Primer 30404 (5′-ACT GAC CTT TAT ATA CGC GCT TG-3′).
- Primer oIMR2088 (5′-AGA CTG CCT TGG GAA AAG CG-3′).

Two PCR reactions were set up:

Reaction A targeted a 200 bp segment of the *Smad4* gene’s wild-type allele using primers 30403 and 30404.

Reaction B aimed to amplify a 300 bp fragment indicative of the *Smad4* knockout allele with primers 30404 and oIMR2088.

Both reactions constitute a touchdown phase. Afterward, the PCR resumed with denaturation at 94.0 °C, annealing at 60.0 °C, and extension at 72.0 °C for 30 cycles. Finally, an extension step was conducted at 72.0 °C, followed by a hold step at 10.0 °C [36].

### 4.4. Phenotyping Protocols

#### 4.4.1. Body Weight Monitoring

Body weights were recorded weekly from weaning (week 4) until 80 weeks of age. Changes in body weight and percentage body weight change over the study period were calculated and used as phenotypic variables for subsequent principal component analysis (PCA).

#### 4.4.2. Organ Weight Measurement and Adjustment

At necropsy, the following organs were excised and weighed: liver, spleen, kidneys, brain and heart. Organ weights were normalized to terminal body weight as follows:

Adjusted Organ Weight (%) = Organ weight (g)/Terminal body weight (g) × 100.

### 4.5. Tissue Processing and Polyp Assessment

#### 4.5.1. Intestinal Collection and Segmental Dissection

Mice were euthanized at 80 weeks or upon reaching endpoint criteria using CO_2_ asphyxiation. Intestines were excised, flushed with PBS, and divided into four anatomical segments: duodenum, jejunum, ileum and colon. These were further grouped into: small intestine (SB1–SB3), colon and total intestinal tract.

#### 4.5.2. Whole-Mount Staining and Polyp Classification

Tissues were fixed in 10% neutral buffered formalin for 24 h and stained with methylene blue [18]. Polyps were counted under a stereo microscope and categorized by size: A: >3 mm, B: 1–3 mm and C: <1 mm. Counts were recorded for each intestinal segment. Prior to analysis, the distributions of the intestinal polyp count variables were assessed for normality. Where necessary, logarithmic transformation was applied to improve normality before inclusion in the subsequent analyses.

### 4.6. Principal Component Analysis (PCA)

Before PCA, all the phenotypic variables were standardized (mean-centered and scaled to unit variance). The distributions of the intestinal polyp count variables were assessed for normality, and logarithmic transformation was applied where necessary before standardization and PCA.

To reduce dimensionality and identify the major axes of phenotypic variation, PCA was applied to 11 standardized phenotypic variables, including delta body weight, adjusted organ weights, and intestinal polyp counts (small intestine, colon, and total).

PCA was conducted in R v4.3.1 using the prcomp() function. Seven principal components (PCs) with eigenvalues > 1 were retained based on the scree plot and cumulative variance explained (>85%). PCA loadings were examined to facilitate the biological interpretation of each component. The resulting PC scores were subsequently used as phenotypic traits for QTL analysis. The loading matrices for the retained principal components are provided in Supplementary Tables S3–S5.

### 4.7. QTL Mapping and Genetic Analysis

#### 4.7.1. Genotyping and Founder Mosaics

High-density SNP genotyping for the Collaborative Cross (CC) founders was performed using the Mouse Diversity Array (MDA), Mouse Universal Genotyping Array (MUGA), and MegaMUGA platforms, comprising approximately 620,000, 7800, and 77,800 SNP markers, respectively [42]. These genome-wide markers are distributed across all the autosomes, the sex chromosomes, and the mitochondrial genome, providing dense genomic coverage for founder haplotype reconstruction. Founder haplotype mosaics were reconstructed using R/qtl2, and ancestral haplotype contributions were inferred from the Collaborative Cross genome reference resources (The Jackson Laboratory) [43].

#### 4.7.2. QTL Analysis with PCA Scores

QTL scans were performed using R/qtl2 with Haley–Knott regression with the leave-one-chromosome-out (LOCO) approach to account for population structure. Principal component (PC) scores were regressed on founder haplotype genotype probabilities across the genome. Genome-wide significance thresholds were determined using 10,000 permutation tests, with significant and suggestive thresholds defined at genome-wide *p* < 0.05 and *p* < 0.10, respectively. QTL support intervals were defined using the classical 1.5-LOD-drop method and were used to identify candidate genes within each QTL region.

#### 4.7.3. Founder Effect Estimation

At each QTL peak, founder strain effect sizes were estimated. Founder contributions were normalized to the WSB/EiJ reference. Merge analysis identified founder-specific alleles driving trait variation.

#### 4.7.4. Candidate Gene Identification

All the annotated genes located within the 1.5-LOD support interval were retrieved using the Mouse Genome Informatics (MGI) database and SNPTools. Gene annotation was based on the *Mus musculus* reference genome (GRCm38/mm10) available through these resources. Retrieved features included protein-coding genes, long noncoding RNAs (lncRNAs), and predicted regulatory elements. Candidate genes were prioritized based on positional overlap with the QTL interval and their potential relevance to intestinal homeostasis, growth, and tumorigenesis.

### 4.8. Statistical Analysis

All the statistical analyses were performed using SPSS v19 and R v4.3.1. ANOVA was used to assess the effects of strain and sex on phenotype variation. PCA and QTL analysis were performed as described above. All the tests were two-tailed, with statistical significance set at *p* < 0.05 unless otherwise noted. Genome-wide significance thresholds for each principal component (PC) were determined by 10,000 permutation tests. QTLs were considered significant if they reached a genome-wide significance level of *p* < 0.05 and suggestive if they reached *p* < 0.1. These significance levels corresponded to specific LOD score thresholds determined for each genome scan.

## 5. Conclusions

This study delivers a comprehensive systems-genetic dissection of intestinal tumor risk and systemic physiology in *Smad*4^+^/^−^ × CC-F1 mice by leveraging Principal Component Analysis and genome-wide QTL mapping. Through this multivariate lens, we identified biologically coherent trait clusters—linking tumor burden, metabolic trends, and organ morphology—and mapped them to both structural and regulatory loci.

Significant QTLs across Chromosomes 1, 4, and 10 reflect the polygenic and pleiotropic nature of *Smad*4-driven phenotypes. Founder-effect and candidate gene analyses emphasized contributions from CAST/EiJ, 129S1/SvImJ, and WSB/EiJ strains, as well as key loci like Galnt7 and Galntl6. Enhancer-rich, noncoding regions and lncRNAs (e.g., Gm11923, Gm40646) also emerged as likely contributors.

Importantly, sex-specific associations underscore the critical role of sex in shaping genetic architecture—particularly in traits related to organ scaling and gut biology. Overall, these results provide a foundational framework for multivariate QTL discovery in genetically diverse models and establish testable hypotheses for how genetic diversity modulates cancer susceptibility and systemic physiology.

By mapping these interactions, this study advances our understanding of complex disease variation and offers translational insights for human colorectal and juvenile polyposis syndromes.

## Figures and Tables

**Table 1:** Genome-wide significant (*p* < 0.05) QTLs detected in combined and sex-specific analyses. Summary of significant loci detected for PCs 1 and 5. The table lists the chromosome, peak position (in megabases), LOD score, phenotypic component, sex, associated trait category, and nearest candidate genes. These loci define the major genetic determinants of the tumor load, as well as the metabolic and organ-scaling axes identified in the study.

Trait Name	Chromosome	QTL Name	Peak (Mb)	95% CI (Mb)	Size (Mb)
Average of PC1	1	*IPSL1*	43.91	43.86–43.95	0.09
Average of PC1	4	*IPSL14*	29.77	29.37–29.83	0.47
Average of PC1 Males	1	*IPSL1*	43.95	43.91–44.01	0.1
Average of PC1 Males	4	*IPSL14*	30.67	30.62–30.67	0.05
Average of PC5 Females	10	*IPSL54*	1.93	0.07–1.94	1.87

**Table 2:** Suggestive (*p* < 0.1) QTLs detected across principal components 1–6. Summary of loci exceeding the 0.1 suggestive threshold. Columns include chromosome, peak position (Mb), LOD score, principal component, sex, associated traits, and biological interpretation. These regions may contain secondary or polygenic modifiers influencing body-weight, organ-size, or polyp-related variation.

Trait Name	Chromosome	QTL Name	Peak (Mb)	95% CI (Mb)	Size (Mb)
Average of PC7 Males	11	*IPSL82*	31.2	31.16–31.21	0.06
Average of PC2 Females	10	*IPSL17*	51.83	51.83–51.83	0.0
Average of PC2 Females	8	*IPSL33*	58.6	58.10–58.64	0.54
Average of PC5 Females	1	*IPSL53*	46.35	18.75–46.47	27.72
Average of PC5 Females	3	*IPSL65*	18.2	18.17–18.22	0.05

## Data Availability

The datasets generated and analyzed during the current study are available at 10.6084/m9.figshare.31986828.

## Supplementary Materials

The following supporting information can be downloaded at https://www.mdpi.com/article/10.3390/ijms27167254/s1.

## Abbreviations

The following abbreviations are used in this manuscript:

CC	Collaborative Cross
QTL	Quantitative trait locus
PCA	Principal component analysis
CRC	Colorectal cancer
IACUC	Institutional Animal Care and Use Committee
SPF	Specific pathogen-free
PBS	Phosphate-buffered saline
AUC	Area under the curve
LOD	Logarithm of the odds
SNP	Single-nucleotide polymorphism
MGI	Mouse Genome Informatics
lncRNA	Long noncoding RNA
ANOVA	Analysis of variance
Ipsl	Intestinal polyposis susceptibility locus
