# Supplementary material for: Integrative Principal Component–QTL Mapping Identifies Genetic Modifiers of Tumor and Metabolic Traits in Smad4-Deficient Collaborative Cross Mice
Source: Int J Mol Sci. 2026 Aug 14;27(16):7254. doi: 10.3390/ijms27167254 (PMC13512919; doi:10.3390/ijms27167254)
Supplement: Supplementary file 1 [file ijms-27-07254-s001.zip › ijms-4342660-supplementary.pdf]

Supplementary Materials

Figure S1. Principal component analysis (PCA) of combined population phenotypes

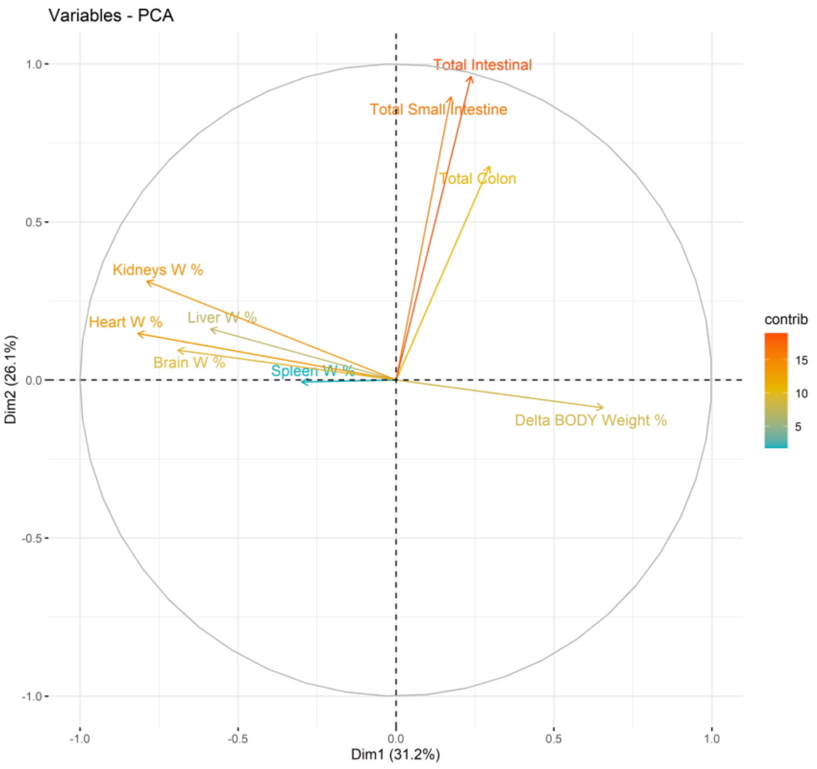

Figure S2. Principal component analysis (PCA) of male *Smad4*$^{+/-}$ × CC-F1 mice

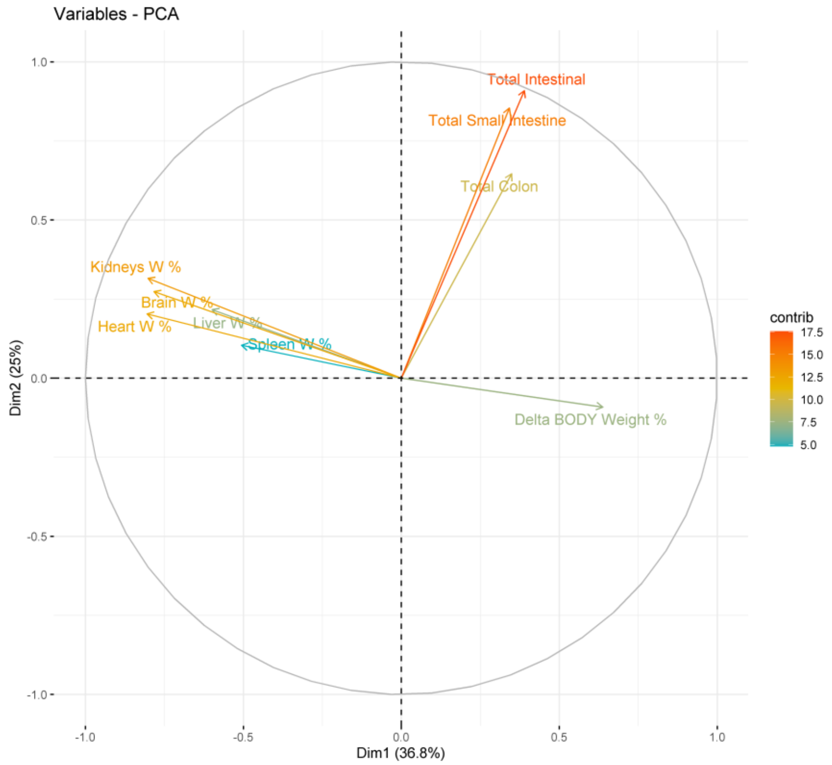

Figure S3. Principal component analysis (PCA) of female *Smad4*$^{+/-}$ × CC-F1 mice

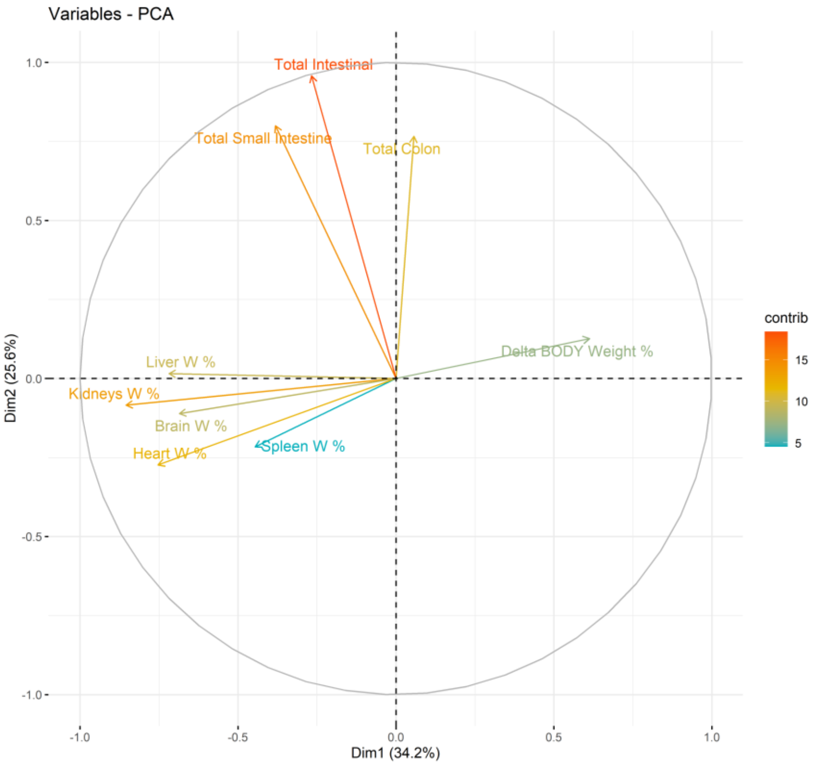

Table S1. QTL detected at exploratory threshold for polyp development across different intestinal segments

Trait Name	Chromosome	QTL Name	Peak (Mb)	95% CI (Mb)	Size (Mb)
Average of PC1	11	Ips3	78.47	78.47–78.83	0.36
Average of PC1	2	Ips12	53.12	53.12–53.16	0.04
Average of PC1	8	Ips18	60.07	60.04–60.14	0.09
Average of PC1	X	Ips20	3.29	1.79–3.29	1.5
Average of PC2	19	Ips31	45.38	45.33–49.09	3.76
Average of PC2	7	Ips37	86.7	86.26–86.75	0.5
Average of PC5	5	Ips64	21.23	21.10–21.29	0.19
Average of PC5	X	Ips68	63.17	63.16–63.24	0.08
Average of PC6	5	Ips81	12.25	10.66–12.26	1.59
Average of PC7	15	Ips90	32.43	30.83–32.62	1.79
Average of PC7	2	Ips92	1.82	0.14–99.82	99.68
Average of PC8	1	Ips99	0.98	0.95–1.00	0.04
Average of PC8	10	Ips100	69.46	67.18–70.46	3.28
Average of PC8	11	Ips101	69.65	68.42–69.81	1.39
Average of PC8	13	Ips102	61.94	56.47–62.99	6.52
Average of PC8	15	Ips103	0.31	0.30–0.32	0.02
Average of PC8	18	Ips106	27.41	27.40–27.47	0.08
Average of PC8	2	Ips108	77.29	77.28–77.46	0.18
Average of PC8	4	Ips110	18.59	18.54–18.60	0.06
Average of PC8	7	Ips113	47.45	47.45–47.45	0.0
Average of PC8	X	Ips114	55.03	46.45–60.10	13.65

Average of PC9	1	Ips115	16.88	6.47–16.89	10.42
Average of PC9	11	Ips117	46.07	44.81–46.18	1.37
Average of PC9	12	Ips118	43.97	41.94–43.97	2.03
Average of PC9	16	Ips122	26.06	26.05–26.64	0.59
Average of PC9	3	Ips127	11.58	11.41–11.80	0.39
Average of PC9	4	Ips128	77.31	2.60–77.50	74.89
Average of PC9	7	Ips131	86.7	13.17–86.75	73.58
Average of PC9	8	Ips132	63.81	22.18–63.84	41.66
Average of PC1 Males	1	Ips1	43.95	43.91–44.01	0.1
Average of PC1 Males	2	Ips12	53.1	53.10–53.11	0.01
Average of PC1 Males	4	Ips14	30.67	30.62–30.67	0.05
Average of PC1 Males	9	Ips19	35.59	35.58–36.45	0.87
Average of PC3 Males	10	Ips42	46.86	46.85–46.87	0.02
Average of PC3 Males	11	Ips43	54.16	43.36–54.16	10.81
Average of PC3 Males	18	Ips50	34.13	34.09–34.53	0.44
Average of PC3 Males	2	Ips52	54.56	54.56–54.56	0.0
Average of PC3 Males	3	Ips53	9.08	9.04–9.27	0.23
Average of PC3 Males	7	Ips57	47.12	46.53–47.12	0.59
Average of PC3 Males	X	Ips60	24.15	24.11–24.21	0.1
Average of PC7 Males	1	Ips80	56.98	56.98–56.98	0.0
Average of PC7 Males	10	Ips81	11.03	10.78–26.50	15.71
Average of PC7 Males	11	Ips82	31.2	31.16–31.21	0.06
Average of PC7 Males	16	Ips87	35.92	35.08–36.01	0.93

Average of PC7 Males	2	Ips89	44.67	44.61–46.42	1.81
Average of PC7 Males	3	Ips90	39.25	39.22–39.26	0.04
Average of PC7 Males	4	Ips91	16.9	16.90–16.91	0.01
Average of PC1 Females	X	Ips15	10.1	10.04–10.15	0.11
Average of PC2 Females	1	Ips16	46.35	46.35–46.35	0.0
Average of PC2 Females	10	Ips17	51.83	51.83–51.83	0.0
Average of PC2 Females	12	Ips19	38.72	38.52–49.17	10.66
Average of PC2 Females	13	Ips20	50.0	49.88–61.38	11.5
Average of PC2 Females	16	Ips23	39.5	38.96–51.47	12.5
Average of PC2 Females	2	Ips27	55.41	55.30–55.44	0.14
Average of PC2 Females	3	Ips28	34.01	33.88–34.05	0.17
Average of PC2 Females	4	Ips29	6.31	6.29–77.31	71.02
Average of PC2 Females	7	Ips32	34.58	34.56–34.59	0.03
Average of PC2 Females	8	Ips33	58.6	58.10–58.64	0.54
Average of PC5 Females	1	Ips53	46.35	18.75–46.47	27.72
Average of PC5 Females	10	Ips54	1.93	0.07–1.94	1.87

Average of PC5 Females	12	Ips56	36.99	36.98–36.99	0.01
Average of PC5 Females	16	Ips60	26.43	26.43–26.51	0.08
Average of PC5 Females	17	Ips61	48.43	48.43–53.12	4.69
Average of PC5 Females	19	Ips63	38.06	35.31–44.06	8.75
Average of PC5 Females	2	Ips64	26.38	25.95–47.37	21.42
Average of PC5 Females	3	Ips65	18.2	18.17–18.22	0.05
Average of PC5 Females	6	Ips68	41.04	40.96–41.14	0.17
Average of PC5 Females	X	Ips72	34.11	34.04–34.13	0.09
Average of PC6 Females	1	Ips73	36.58	0.68–36.59	35.92
Average of PC6 Females	16	Ips79	54.64	54.64–54.68	0.04
Average of PC6 Females	6	Ips85	48.4	46.20–48.40	2.21
Average of PC6 Females	9	Ips88	37.12	36.85–37.33	0.48
Average of PC6 Females	X	Ips89	35.81	35.74–35.85	0.11
Average of PC7 Females	11	Ips90	68.14	68.12–68.14	0.02
Average of PC7 Females	3	Ips94	65.42	38.80–65.54	26.74

Average of PC7 Females	5	Ips96	39.9	39.06–55.47	16.41
Average of PC7 Females	8	Ips98	55.52	52.15–55.79	3.63

Table S2: A summary of the sample size of male and female mice used from the 14 different line backgrounds of the Collaborative Cross mouse population.

F1 (Samd4^{KO} X CCxxx)	TAU CC Lines			Sex		Total
				F	M	
CC037	IL72	Genotype	WT	17	15	32
			KO	14	19	33
		Total		31	34	65
CC004	IL188	Genotype	WT	4	5	9
			KO	11	8	19
		Total		15	13	28
CC040	IL557	Genotype	WT	1	2	3
			KO	1	2	3
		Total		2	4	6
-	IL711	Genotype	WT	4	4	8
			KO	3	3	6
		Total		7	7	14
CC019	IL2513	Genotype	WT	4	3	7
			KO	12	4	16

		Total		16	7	23
CC006	IL2750	Genotype	WT	7	5	12
			KO	10	7	17
		Total		17	12	29
-	IL3348	Genotype	WT	12	12	24
			KO	13	11	24
		Total		25	23	48
CC059	IL3912	Genotype	WT	4	9	13
			KO	4	6	10
		Total		8	15	23
CC041	IL4141	Genotype	WT	2	0	2
			KO	2	0	2
		Total		4		4
CC010	IL5000	Genotype	WT	11	12	23
			KO	14	10	24
		Total		25	22	47
CC018	IL5008	Genotype	WT	16	8	24
			KO	14	15	29
		Total		30	23	53
-	IL5012	Genotype	WT	7	4	11
			KO	5	6	11
		Total		12	10	22
CC035	IL6009	Genotype	WT	11	8	19
			KO	5	13	18

		Total		16	21	37
-	IL6012	Genotype	WT	9	11	20
			KO	8	11	19
		Total		17	22	39
-	IL6018	Genotype	WT	20	12	32
			KO	15	14	29
		Total		35	26	61
Total		Genotype	WT	129	110	239
			KO	131	129	260
		Total		260	239	499

Table S3 PCA loading matrix for the combined dataset. Loadings of the nine standardized phenotypic var-iables on the seven retained principal components (PC1–PC7). Positive and negative values indicate the direction and magnitude of each variable's contribution to the corresponding principal com-ponent

Phenotypic Variable	PC1	PC2	PC3	PC4	PC5	PC6	PC7
Total Small Intestine	0.104	0.585	0.061	0.138	-0.462	0.113	0.086
Total Colon	0.177	0.442	-0.053	-0.142	0.761	-0.277	-0.164
Total Intestinal	0.142	0.627	0.035	0.072	-0.151	0.008	0.022
Liver W (%)	-0.350	0.106	-0.204	-0.696	-0.309	-0.190	-0.431
Spleen W (%)	-0.178	-0.004	0.910	0.049	-0.019	-0.241	-0.272
Kidneys W (%)	-0.471	0.204	0.007	-0.161	0.238	0.410	0.096
Heart W (%)	-0.488	0.096	0.168	-0.088	0.141	0.207	0.511
Brain W (%)	-0.412	0.062	-0.224	0.297	-0.078	-0.746	0.290
Delta Body Weight (%)	0.391	-0.057	0.208	-0.589	-0.072	-0.221	0.592

Table S4 PCA loading matrix for female mice

Phenotypic Variable	PC1	PC2	PC3	PC4	PC5	PC6	PC7
Total Small Intestine	-0.218	0.527	0.012	0.200	-0.483	-0.236	0.121
Total Colon	0.032	0.505	0.114	-0.208	0.697	0.291	-0.059
Total Intestinal	-0.152	0.631	0.061	0.060	-0.057	-0.050	0.066
Liver W (%)	-0.410	0.010	-0.207	-0.526	-0.336	0.443	-0.428
Spleen W (%)	-0.254	-0.142	0.701	0.044	-0.145	0.470	0.406
Kidneys W (%)	-0.487	-0.056	0.139	0.099	0.176	-0.290	-0.385
Heart W (%)	-0.429	-0.180	0.306	-0.104	0.235	-0.448	-0.052
Brain W (%)	-0.391	-0.073	-0.475	-0.288	0.162	-0.085	0.688
Delta Body Weight (%)	0.349	0.083	0.330	-0.729	-0.176	-0.385	0.073

Table S5 PCA loading matrix for male mice

Phenotypic Variable	PC1	PC2	PC3	PC4	PC5	PC6	PC7
Total Small Intestine	0.188	0.569	0.043	-0.097	0.473	-0.011	-0.030
Total Colon	0.192	0.430	0.019	0.123	-0.824	0.050	0.045
Total Intestinal	0.214	0.605	0.042	-0.048	0.172	0.004	-0.013
Liver W (%)	-0.329	0.144	-0.506	0.471	0.140	0.577	0.032
Spleen W (%)	-0.278	0.069	0.774	0.540	0.073	0.048	0.134
Kidneys W (%)	-0.440	0.210	-0.118	0.051	-0.169	-0.162	-0.546
Heart W (%)	-0.442	0.135	-0.072	-0.018	0.067	-0.565	-0.177

Phenotypic Variable	PC1	PC2	PC3	PC4	PC5	PC6	PC7
Brain W (%)	-0.430	0.182	-0.132	-0.242	-0.069	-0.147	0.783
Delta Body Weight (%)	0.350	-0.061	-0.322	0.631	0.069	-0.543	0.187
